# Common cardiovascular biomarkers can independently predict outcome of patients with Myelodysplastic syndromes

**DOI:** 10.1038/s41408-023-00844-4

**Published:** 2023-05-03

**Authors:** Ioannis Mitroulis, Vasileios Papadopoulos, Eleftheria Lamprianidou, Peter Mirtschink, Konstantinos Liapis, Kalliopi Zafeiropoulou, Alexandra Kourakli, Theodoros Moysiadis, Menelaos Papoutselis, George Vrachiolias, Argiris Symeonidis, Ioannis Kotsianidis

**Affiliations:** 1 Department of Hematology, Democritus University of Thrace Medical School, Alexandroupolis, Greece; 2 Institute for Clinical Chemistry and Laboratory Medicine, University Hospital and Faculty of Medicine Carl Gustav Carus of TU Dresden, Dresden, Germany; 3 Hematology Division, Department of Internal Medicine, University of Patras Medical School, Patras, Greece; 4 Department of Computer Science, School of Sciences and Engineering, University of Nicosia, Nicosia, 2417 Cyprus

**Keywords:** Risk factors, Translational research

Dear Editor,

Myelodysplastic syndromes (MDS) comprise a heterogeneous group of clonal myeloid disorders characterized by ineffective hematopoiesis and varying degrees of leukemic transformation [1]. Several well-validated prognostic systems have been developed to help clinicians in predicting the disease course and design evidence-based treatment strategies [2]. However, in particular for lower-risk MDS, the prognosis assessment remains problematic as clinical strategies range from watchful waiting to early allogeneic stem cell transplantation [3]. In addition, with the exception of cytogenetics and only very recently somatic mutations [4], no other biomarkers have been incorporated in the algorithm of MDS prognosis, reflecting the largely uncharted pathobiology and heterogeneous course of MDS.

It has been reported that soluble biomarkers of cardiovascular disease (CVD) such as N-terminal pro-B-type natriuretic peptide (NT-proBNP) and growth differentiation factor-15 (GDF-15) may drive tumor growth and are apparently linked to cancer incidence [5, 6]. Conversely, the decrease of high-sensitivity C-reactive protein (Hs-CRP) levels after statin treatment unexpectedly resulted in a significant reduction of cancer mortality [7], while NT-proBNP and troponin T (TNT) levels were independently associated with all-cause mortality in patients with various malignancies, irrespective of the presence or not of CVD [8]. MDS typically affect elderly individuals carrying several comorbidities which can considerably influence clinical outcome and most patients succumb to conditions unrelated to MDS, with cardiovascular disease being the second most common cause of non-MDS-related mortality [9, 10]. On top of epidemiological evidence, experimental data further support a pathophysiological link between clonal hematopoiesis of indeterminate potential (CHIP), a precursor of MDS, with CVD development through inflammation-mediated accelerated atherosclerosis [11, 12]. Despite the evidence of a bidirectional interplay between CVD and malignancies and the reported association of CVD biomarkers with all-cause mortality in cancer patients no study to date has addressed the value of CVD biomarkers in the prognostic assessment of MDS patients.

We performed a multicenter retrospective cohort study that included 105 patients with MDS. Serum levels of TNT, proBNP, GDF-15, and CRP, were measured in all patients. Survival analysis was performed using Kaplan–Meier estimates and multivariate analysis by using Cox regression. Overall survival (OS) was defined as the time from sampling to last follow-up or death from any cause and Leukemia-free survival (LFS) as the time from sampling to leukemic progression or death. Time to progression (TTP) was defined as the time from sampling to the date of disease progression. Details of the statistical analysis are presented in Supplemental Methods. The study was approved by the institutional review boards and it was performed in compliance with the Declaration of Helsinki.

Demographic and disease characteristics are summarized in Table 1. With a median follow-up of 23.9 (95% CI: 11–36.8) months the median OS and TTP for the whole cohort was 37 (95% CI: 12.6–61.4) and 25 (95% CI: 11.9–38.1) months, respectively. Progression to AML was observed in 36 (34.3%) patients and CVD was the primary cause for 3/30 (10%) of reported deaths.

**Table 1:** Characteristics of MDS patients and univariate and multivariate analysis for overall survival (OS) and time to progression (TTP).

Parameters	Median (Range)^a^, *N* (%)^b^	TTP Univariate analysis *P*-value	TTP Univariate analysis HR; 95%CI	TTP Multivariate analysis *P*-value^c^	TTP Multivariate analysis HR; 95%CI	OS Univariate analysis *P*-value	OS Univariate analysis HR; 95%CI	OS Multivariate analysis *P*-value^c^	OS Multivariate analysis HR; 95%CI
*Age*									
Median (Range)	73 (20–89)	0.389	1.146 (0.840–1.565) per every 10 years >72			0.383	1.194 (0.801–1.780) per every 10 years >72		
*Sex*									
Males	72 (68.6)	0.071	1.898 (0.947–3.802) for males			0.184	1.761 (0.764–4.065) for males		
Females	33 (31.4)								
*Hb*									
Median (Range)	9.7 (7.0–15.5)	**0.041**	0.835 (0.702–0.993) for every unit >10			**0.040**	0.795 (0.639–0.989) for every unit >10		
N/A	1								
*ANC (x1000)*									
Median (Range)	2.0 (0.0–19.7)	**0.020**	1.040 (1.006–1.075) for every unit >4			0.123	1.036 (0.990–1.084) for every unit >4		
N/A	1								
*PLT (x1000)*									
Median (Range)	148 (8–770)	0.159	0.836 (0.651–1.073) for every 100 units >165			0.201	0.814 (0.595–1.115) for every 100 units >165		
N/A	1								
*BM blasts*									
Median (Range)	3.0 (0.0–86.0)	**0.005**	1.034 (1.010–1.058) for every unit >7	**0.001**	1.041 (1.017–1.066) for every unit >7	**0.001**	1.042 (1.017–1.068) for every unit >7	**<0.001**	1.054 (1.027–1.081) for every unit >7
N/A	1								
*Cytogenetics (IPSS-R)*									
Very good	12 (12.0)	**0.040**	1.871 (1.028–3.407) for Cytogenetics IPSS-R risk categories Intermediate/High/Very High			0.209	1.602 (0.768–3.341) for Cytogenetics IPSS-R risk categories Intermediate/High/Very High		
Good	63 (63.0)								
Intremediate	15 (15.0)								
Poor	5 (5.0)								
Very poor	5 (5.0)								
N/A	5								
*Mutations (total)*									
Median (Range)	1.0 (0.0–6.0)	**0.042**	1.388 (1.012–1.904) for every unit >1	Ν/Α	Ν/Α	**0.032**	1.042 (1.017–1.068) for every unit >1	Ν/Α	Ν/Α
N/A	74								
*MDS-CI risk category*									
Low	70 (68.6)	0.442	0.794 (0.442–1.429) for every risk category higher than “Low”			0.790	1.079 (0.617–1.889) for every risk category higher than “Low”		
Intermediate	25 (24.5)								
High	7 (6.9)								
N/A	3								
*Transfusion dependence*									
YES	70 (30.0)								
NO	30 (30.0)	0.351	1.344 (0.722–2.504) for transfusion dependence			0.415	1.361 (0.649–2.584) for transfusion dependence		
N/A	5								
*CRP (mg/l)*									
Median (Range)	4.1 (0.0–128.0)	**0.030**	1.109 (1.010–1.218) for every ten units >15			**0.003**	1.158 (1.050–1.278) for every ten units >15		
N/A	2								
*Troponin (ng/l)*									
Median (Range)	14.0 (0.0–193.0)	0.940	0.993 (0.833–1.184) for every ten units >20			0.374	1.071 (0.921–1.245) for every ten units >20		
N/A	1								
*GDF-15 (ng/l)*									
Median (Range)	3184 (444–32,043)	**0.011**	1.062 (1.014–1.112) for every 1000 units >5500	**0.017**	1.063 (1.011–1.118) for every 1000 units >5500	**0.010**	1.075 (1.015–1.135) for every 1000 units >5500	**0.015**	1.080 (1.015–1.149) for every 1000 units >5500
N/A	1								
*NT-proBNP (ng/l)*									
Median (Range)	41.6 (2.1–1614)	**0.006**	1.201 (1.055–1.368) for every 100 units >110	**0.007**	1.207 (1.052–1.384) for every 100 units >110	**0.001**	1.246 (1.099–1.412) for every 100 units >115	**0.001**	1.270 (1.107–1.457) for every 100 units >110
N/A	1								

*IPSS* International Prognostic Scoring System, *IPSS-R* revised International Prognostic Scoring System, *AML* acute myeloid leukemia, *NA* not applicable (missing).

^a^For continuous variables.

^b^For discrete variables.

^c^Initial model included all statistically significant variables in univariate analysis and reduced step-by-step (*P*_IN_ = 0.05; *P*_OUT_ = 0.10); HR > 1 indicates unfavorable effect, while HR < 1 favorable effect.

Bold values identify statistical significance.

Univariate and multivariate analyses at diagnosis are presented at Table 1. Baseline levels of hemoglobin, percentage of bone marrow blasts (BMΒ) and CRP, GDF-15, and NT-proBNP levels were identified as significant prognosticators for OS, whereas hemoglobin, ANC, BMB cytogenetic category, CRP, NT-proBNP, and GDF-15 were significantly associated with TTP in univariate analysis. In multivariate analysis only BMB (HR = 1.054, 95% CI = 1.027–1.081), GDF-15 (HR = 1.080, 95% CI = 1.015–1.149), and NT-proBNP (HR = 1.270, 95% CI = 1.107–1.457) correlated independently with OS, while the same parameters, BMB (HR = 1.041, 95% CI = 1.017–1.066), GDF-15 (HR = 1.063, 95% CI = 1.011–1.118), and NT-proBNP (HR = 1.207, 95% CI = 1.052–1.384) were also independently associated with TTP.

In order to assess the power of GDF-15 and NT-proBNP as standalone prognosticators we determined the best cutoff levels for each parameter using maximally selected rank statistics. A GDF-15 value above the cutoff of 3727 ng/L was significantly associated with worse OS (*p* = 0.010) and TTP (*p* = 0.006, Supplemental Fig. 1), whereas the selected value for NT-proBNP (175 ng/L) could not act as a prognosticator for either OS or TTP (Supplemental Fig. 2).

We then addressed the prognostic power of the combination of these two cardiac markers by constructing a composite score (CardioScore) with the values of each NT-proBNP or GDF-15 above the aforementioned cutoff scored with 0.5. Cardioscore stratified patients in 3 categories (0, 0.5, and 1 point) with significantly different OS (*p* = 0.010) and TTP (*p* = 0.043, Fig. 1A, B) after adjustment for age and gender. We further evaluated whether combining IPSS-R [13], currently the most widely used prognostic system, with CardioScore could improve the prognostic power of the former. The combined IPSS-RC score, defined as the sum of IPSS-R score and CardioScore (Supplemental Table 1) resulted in upstaging of 12/96 (12.5%) of the patients (Fig. 1C) and performed better than IPSS-R, for both OS (*p* = 0.0008; AICc = 212.980 vs *p* = 0.0014; AICc = 214.422, respectively) and TTP (*p* = 0.0008; AICc = 332.735 vs *p* = 0.0018; AICc = 334.031, respectively, Supplemental Table 2).

**Fig. 1:**
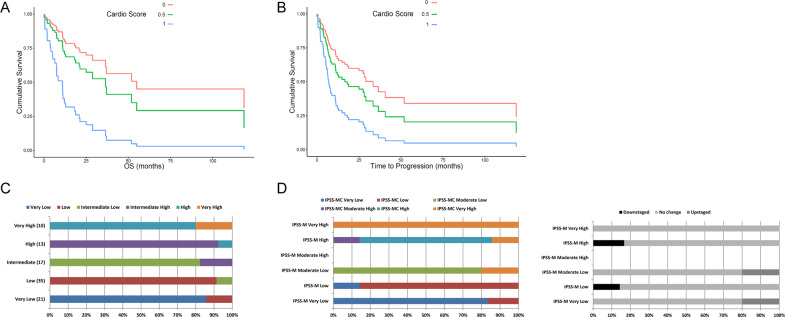
Cardioscore predicts overall survival and time to progression in MDS patients. **A** CardioScore (defined as the sum of 1/2 point if GDF-15 ≥ 3727 ng/L and 0.5 point if NT-proBNP ≥175 ng/L) correlated significantly with OS (*p* = 0.010) after adjustment for age (*p* = 0.382) and gender (*p* = 0.447) using Cox regression (Omnibus test *p* = 0.010). Hazard ratio is 3.086 (95% CI: 1.131–8.403; *p* = 0.028) and 4.831 (95% CI: 1.748–13.35; *p* = 0.002) for CardioScore = 0.5 and 1, respectively, when compared with baseline hazard (HR = 1.000; CardioScore = 0). **B** Cardioscore also correlated significantly with TTP (*p* = 0.043), after adjustment for age (*p* = 0.542) and gender (*p* = 0.173) using Cox regression (Omnibus test *p* = 0.032). HR is 2.012 (95% CI: 0.831–4.854; *p* = 0.121) and 3.030 (95% CI: 1.253–7.299; *p* = 0.014) for CardioScore = 0.5 and 1, respectively, when compared with baseline hazard (HR = 1.000; CardioScore = 0). **C** Restratification of IPSS-R to IPSS-RC, the latter defined as the sum of IPSS-R score and CardioScore. IPSS-RC upstaged 12.5% of MDS patients. **D** Restratification of IPSS-M to IPSS-MC, a composite score constructed by integrating the levels of NT-proBNP into IPSS-M. IPSS-RC redistributed 20.7% of MDS patients.

The advent of molecular analysis led to the implementation of a molecular prognostic model for MDS, the IPSS-M [4]. To question whether the CVD biomarkers still hold their independent prognostic power when somatic mutations are included in the prognostic algorithm we performed a separate analysis in the selected group of patients with available mutational data (*n* = 29, Supplemental Table 3). In multivariate analysis the levels of NT-proBNP were independently associated with TTP (HR = 1.586, 95% CI = 1.139–2.207; *p* = 0.006) and OS (HR = 2.023, 95% CI = 1.109–3.690; *p* = 0.022) along with the number of identified mutations and BMB. We then used Cox regression to generate IPSS-MC score, a NT-proBNP adjusted IPSS-M score, which redistributed 6/29 (20.7%) of the patients (Fig. 1D) and performed better than IPSS-M, for both OS (*p* = 0.0006; AICc = 41.566 vs *p* = 0.002; AICc = 46.766, respectively) and TTP (*p* = 0.0008; AICc = 70.605 vs *p* = 0.008; AICc = 74.414, respectively (Supplemental Table 4).

Our findings reveal a previously unrecognized association between circulating NT-proBNP and GDF-15 with MDS course and outcome and are in line with numerous reports showing a robust association between cardiac and cancer incidence and mortality [14–16]. Of note, risk factors for CVD and pre-existing CVD, as captured by the MDS-CI index in our analyses, did not affect OS and TTP, indicating that the correlation of survival with CVD biomarkers is rather linked to non-CVD-related or at least all-cause death. We acknowledge that our cohort was limited and the analysis was based only on baseline values not accounting for fluctuations on hematological parameters and CVD biomarkers that cannot be reliably captured retrospectively, whereas the very low number of CVD deaths precludes definite conclusions. However, the independent correlation of NT-proBNP and GDF-15 with TTP agues further for a potential causal association between CVD biomarkers and MDS pathobiology by forming a vicious cycle linking CVD with clonal hematopoiesis [17]. Future studies in large, prospectively annotated, cohorts are needed to definitely address whether this feed forward loop indeed exists and circulating cardiac biomarkers can lead to enhanced risk of progression to myeloid malignancies in individuals with CHIP and/or drive progression in MDS patients.

## Supplementary information

Supplemental methods and tables
