# Supplementary material for: Common cardiovascular biomarkers can independently predict outcome of patients with Myelodysplastic syndromes
Source: Blood Cancer J. 2023 May 3;13(1):64. doi: 10.1038/s41408-023-00844-4 (PMC10156800; doi:10.1038/s41408-023-00844-4)
Supplement: Supplementary file 1 — Supplemental methods and tables [file 41408_2023_844_MOESM1_ESM.docx]

**Supplemental Methods**

**Patients and assays of CVD biomarkers**

Patients with MDS were recruited at the Department of Hematology of the University Hospital of Alexandroupolis and the Department of Hematology of the University Hospital of Patras Greece. The study was approved by the institutional review boards at both participating sites and it was performed in compliance with the Declaration of Helsinki. Levels of troponin T, NT-proBNP, GDF-15 and C-reactive protein (CRP), low-density lipoprotein (LDL-C) and high-density lipoprotein (HDL-C) cholesterol were measured in collected sera using Elecsys® immunoassay kits (ROCHE Diagnostics) at a Cobas (ROCHE Diagnostics) e801 module (hs-TnT, NT-proBNP and GDF-15) or a Cobas c701 module (CRP, HDL-C, LDL-C) respectively according to manufacturer’s instructions.

**Next Generation Sequencing and IPSS-M calculation**

Next Generation sequencing was available in 30 patients and was performed by either an in-house targeted panel (n=8) or by using the Illumina Trusight Myeloid Sequencing Panel (n=13) as described elsewhere[^1^](#_ENREF_1)^,^[^2^](#_ENREF_2). In 9 patients a 19-gene panel was used[^3^](#_ENREF_3). Two patients (6.7%) had no mutations detected and one patient had no cytogenetic data available, thus IPSS-M cannot be calculated. In the remaining 29 patients IPSS-M was estimated using the available IPSS-M Web calculator (https://mds-risk-model.com).

**Statistical analysis**

Calculation of median follow-up time was based on the reverse Kaplan Meier estimator method. Kaplan Meier (KM) curves were used to depict survival data; the log-rank test was used to determine the univariate significance of the study variables. To explore the independent correlations between the four parameters used as markers for cardiovascular disease (CRP, hs-TnT, GDF-15, and NT-proBNP) along with age, gender, MDS-CI category and IPSS-R components (hemoglobin, absolute neutrophil count, platelets, bone marrow blasts, and cytogenetic category), with either OS or TTP, univariate and multivariate analysis were performed using Cox proportional hazards regression analysis; every parameter that was significantly correlated in a certain univariate analysis (p≤0.05) was treated as a potential independent parameter in the relevant multivariate one (the probability for stepwise entry and removal were set to 0.05 and 0.10, respectively; the classification cutoff was set to 0.5; and the maximum number of iterations was set to 20).To turn GDF-15 and NT-pro-BNP variables from scale to binary, the best cutoffs were determined by employing the “maxstat.test” R-function. This function performs tests of independence of a response variable and an independent variable of interest using maximally selected rank statistics, and estimates the appropriate cutoff for the independent variable. Within “maxstat.test”, smethod="LogRank" (log-rank scores-based statistic), and pmethod="condMC"(the p-value approximation was performed by simulating the distribution via conditional Monte-Carlo) were used. The relative quality of statistical models was evaluated using the Akaike information criterion after correction for small samples (AICc).To compare outcomes between two independent groups, the Mann-Whitney U test was preferred in case of n≤30. Correlations between continuous variables were approached by Spearman’s correlation coefficient *ρ* (rho) in case that either outliers were detected or normality was violated in either Kolmogorov-Smirnov or Shapiro-Wilk tests; else, Pearson’s correlation coefficient was alternatively preferred. Benjamini-Hochberg correction was applied when multiple hypotheses were simultaneously tested using the freely available on-line tool at <https://www.sdmproject.com/utilities/?show=FDR>.

IPSS-RC prognostic risk categorization derive from ΙPSS-R risk categorization raised by one level in case that both GDF-15 and NT-proBNP are high (CardioScore =1); its values are the same for both OS and TTP. IPSS-MC score is a NT-proBNP adjusted IPSS-M score, which derives from the relative Cox regression models and thus is specific for either OS or TTP; in detail, IPSS-MC for OS is log2 [(OS-related HR attributable to IPSS-M score)(OS-related HR attributable to NT-proBNP)], while IPSS-MC for TTP is log2[ (TTP-related HR attributable to IPSS-M score)(TTP-related HR attributable to NT-proBNP)] (supplemental table 4).

Descriptive statistics are provided either as medians along with the relevant ranges, or percentages, for scale and discrete parameters respectively. All reported p values are two-sided. The level of statistical significance was set to a=0.05. All numerical values are given with at least two significant digits. Statistical analysis and visualization of KM curves was performed with the use of IBM SPSS Statistics software, version 26.0, for Windows, and the R programming language V4.2.1. The Review Manager version 5.3 (RevMan, Copenhagen: The Nordic Cochrane Centre, The Cochrane Collaboration; 2014) was used to illustrate forest plots.

**Supplemental Table 1**.IPSS-RC prognostic risk categorization derive from IPSS-R risk categorization raised by one level in case that both GDF-15 and NT-proBNP are high (CardioScore =1).

| IPSS-RC risk category | IPSS-R risk score | |
| --- | --- | --- |
|  | GDF-15 <3727 ng/L OR pro-BNP <175 ng/L | GDF-15 ≥3727 ng/L AND pro-BNP ≥175 ng/L |
| Very Low | ≤1.5 | NA† |
| Low | >1.5 - 3 | ≤1.5 |
| Intermediate Low | >3 - 4.5 | >1.5 - 3 |
| Intermediate High | >4.5 – 6 | >3 - 4.5 |
| High | >6 | >4.5 – 6 |
| Very High | NA† | >6 |

† NA: Not applicable

**Suppl. Table 2.** AICc values for models predicting TTP and OS (IPSS-RC vs IPSS-R score); ℓ: maximized value of the likelihood function of the model.

|  | **HR ±95% CI** | **P value** | **k** | **-2ln(ℓ)** | **n** | **AICc** |
| --- | --- | --- | --- | --- | --- | --- |
| **TTP** |  |  |  |  |  |  |
| **IPSS-R** | **1.240 (1.084-1.419)** | **0.0018** | **1** | **333.988** | **94** | **334.031** |
| **IPSS-RC** | **1.247 (1.096-1.419)** | **0.0008** | **1** | **332.692** | **94** | **332.735** |
| **OS** |  |  |  |  |  |  |
| **IPSS-R** | **1.306 (1.109-1.538)** | **0.0014** | **1** | **214.379** | **94** | **214.422** |
| **IPSS-RC** | **1.315 (1.125-1.537)** | **0.0006** | **1** | **212.937** | **94** | **212.980** |

**Supplemental Table 3.**Univariate and multivariate analysis for TTP and OS in patients with available molecular data (n=29)

| **Parameters** | **Median (Range)†,**  **N (%)‡** | **TTP**  **Univariate analysis**  **P-value** | **TTP**  **Univariate analysis**  **HR; 95%CI** | **TTP**  **Multivariate analysis**  **P-value**⁋ | **TTP**  **Multivariate analysis**  **HR; 95%CI** | **OS**  **Univariate analysis**  **P-value** | **OS**  **Univariate analysis**  **HR; 95%CI** | **OS**  **Multivariate analysis**  **P-value**⁋ | **OS**  **Multivariate analysis**  **HR; 95%CI** |
| --- | --- | --- | --- | --- | --- | --- | --- | --- | --- |
| **Age** |  |  |  |  |  |  |  |  |  |
| Median (Range) | 73 (20-89) | 0.988 | 1.146 (0.952-1.052) per every year > 70 |  |  | 0.823 | 0.993 (0.936-1.054) per every year > 70 |  |  |
| **Sex** |  |  |  |  |  |  |  |  |  |
| Males | 72 (68.6) | 0.465 | 1.594 (0.457-5.567) for males |  |  | 0.812 | 1.170 (0.321-4.262) for males |  |  |
| Females | 33 (31.4) |  |  |  |  |  |  |  |  |
| **Hb** |  |  |  |  |  |  |  |  |  |
| Median (Range) | 9.7 (8.6-10.8) | 0.529 | 0.916 (0.697-1.204) for every unit >10 |  |  | 0.288 | 0.838 (0.604-1.161) for every unit >10 |  |  |
| N/A | 1 |  |  |  |  |  |  |  |  |
| **ANC (x1000)** |  |  |  |  |  |  |  |  |  |
| Median (Range) | 2.0 (1.1-3.9) | 0.084 | 1.075 (0.990-1.166) for every unit >4 |  |  | 0.065 | 1.084 (0.995-1.180) for every unit >4 |  |  |
| N/A | 1 |  |  |  |  |  |  |  |  |
| **PLT (x1000)** |  |  |  |  |  |  |  |  |  |
| Median (Range) | 148 (8-770) | 0.067 | 0.995 (0.990-1.000) for every unit >150 |  |  | 0.097 | 0.995 (0.990-1.001) for every unit >150 |  |  |
| N/A | 1 |  |  |  |  |  |  |  |  |
| **BM blasts** |  |  |  |  |  |  |  |  |  |
| Median (Range) | 3.0 (1.3-7.0) | **0.014** | 1.057 (1.011-1.105) for every unit >7 | **0.025** | 1.065 (1.008-1.126) for every unit >7 | **0.001** | 1.109 (1.046-1.175) for every unit >7 | **<0.001** | 1.184 (1.082-1.297) for every unit >7 |
| N/A | 1 |  |  |  |  |  |  |  |  |
| **Cytogenetics (IPSS-R)** |  |  |  |  |  |  |  |  |  |
| Very good | 12 (12.0) | 0.092 | 2.427 (0.865-6.810) for Cytogenetics IPSS-R risk categories Intermediate / High / Very High |  |  | 0.122 | 2.477 (0.784-7.827) for Cytogenetics IPSS-R risk categories Intermediate / High / Very High |  |  |
| Good | 63 (63.0) |  |  |  |  |  |  |  |  |
| Intremediate | 15 (15.0) |  |  |  |  |  |  |  |  |
| Poor | 5 (5.0) |  |  |  |  |  |  |  |  |
| Very poor | 5 (5.0) |  |  |  |  |  |  |  |  |
| N/A | 5 |  |  |  |  |  |  |  |  |
| **Mutations (total)** |  |  |  |  |  |  |  |  |  |
| Median (Range) | 1.0 (0.0-3.0) | **0.042** | 1.388 (1.012-1.904) for every unit >1 | **0.005** | 1.901 (1.215-2.972) for every unit >1 | **0.032** | 1.373 (1.028-1.833) for every unit >1 | **0.003** | 1.941 (1.259-2.922) for every unit >1 |
| N/A | 74 |  |  |  |  |  |  |  |  |
| **MDS-CI risk category** |  |  |  |  |  |  |  |  |  |
| Low | 70 (68.6) | 0.247 | 1.960 (0.628-6.119) for every risk category higher than “Low” |  |  | 0.177 | 2.855 (0.622-13.106) for every risk category higher than “Low” |  |  |
| Intermediate | 25 (24.5) |  |  |  |  |  |  |  |  |
| High | 7 (6.9) |  |  |  |  |  |  |  |  |
| N/A | 3 |  |  |  |  |  |  |  |  |
| **Transfusion dependence** |  |  |  |  |  |  |  |  |  |
| Yes | 30 (30.0) | 0.595 | 1.322 (0.473-3.693) for transfusion dependence |  |  | 0.538 | 1.456 (0.440-4.820) for transfusion dependence |  |  |
| No | 70 (70.0) |  |  |  |  |  |  |  |  |
| N/A | 5 |  |  |  |  |  |  |  |  |
| **CRP (mg/l)** |  |  |  |  |  |  |  |  |  |
| Median (Range) | 4.1 (1.8-15.0) | **0.012** | 1.385 (1.073-1.787) for every ten units >10 |  |  | **0.003** | 1.827 (1.175-2.253) for every ten units >10 |  |  |
| N/A | 2 |  |  |  |  |  |  |  |  |
| **Troponin (ng/l)** |  |  |  |  |  |  |  |  |  |
| Median (Range) | 14.0 (0.0-193.0) | 0.653 | 0.898 (0.562-1.436) for every ten units >20 |  |  | 0.711 | 0.921 (0.530-1.602) for every ten units >20 |  |  |
| N/A | 1 |  |  |  |  |  |  |  |  |
| **GDF-15 (ng/l)** |  |  |  |  |  |  |  |  |  |
| Median (Range) | 3184 (1907-6388) | 0.630 | 1.039 (0.892-1.211) for every 1000 units >3900 |  |  | 0.397 | 1.073 (0.911-1.265) for every 1000 units >3900 |  |  |
| N/A | 1 |  |  |  |  |  |  |  |  |
| **NT-proBNP (ng/l)** |  |  |  |  |  |  |  |  |  |
| Median (Range) | 41.6 (22.4-75.1) | **0.045** | 1.337 (1.007-1.776) for every 100 units >120 | **0.006** | 1.586 (1.139-2.207) for every 100 units >110 | 0.053 | 1.392 (0.995-1.948) for every 100 units >120 | **0.022** | 2.023 (1.109-3.690) for every 100 units >110 |
| N/A | 1 |  |  |  |  |  |  |  |  |

**Abbreviations**: IPSS: International Prognostic Scoring System; IPSS-R: revised International Prognostic Scoring System; NA: Not applicable (missing)

† For continuous variables

‡ For discrete variables

⁋ Initial model included all statistically significant variables in univariate analysis and reduced step-by-step (P_IN_=0.05; P_OUT_=0.10); HR > 1 indicates unfavorable effect, while HR <1 favorable effect.

**Supplemental Table 4.** Models predicting TTP and OS based on IPSS-M score, IPSS-M score adjusted for NT-proBNP values, and IPSS-MC score; ℓ: maximized value of the likelihood function of the model.

|  | **B** | **SE** | **Covariate mean** | **HR ±95% CI** | **P value** | **k** | **-2ln(ℓ)** | **n** | **AICc** |
| --- | --- | --- | --- | --- | --- | --- | --- | --- | --- |
| **TTP** |  |  |  |  |  |  |  |  |  |
| **IPSS-M score** | 0.518 | 0.196 | -0.197 | 1.679 (1.144-2.463) | 0.008 | 1 | 74.414 | 28 | 76.568 |
| **IPSS-M score**  **NT-proBNP (per 100 increment)** | 0.508  0.294 | 0.196  0.139 | -0.197  1.211 | 1.662 (1.131-2.442)  1.342 (1.021-1.763) | 0.010  0.035 | 2 | 70.428 | 28 | 74.908 |
| **IPSS-MC score (exact)†** | 0.482 | 0.144 | -0.001 | 1.619 (1.220-2.148) | 0.0008 | 1 | 70.605 | 28 | 72.759 |
| **OS** |  |  |  |  |  |  |  |  |  |
| **IPSS-M score** | 0.765 | 0.251 | -0.197 | 2.149 (1.314-3.514) | 0.002 | 1 | 46.766 | 28 | 48.920 |
| **IPSS-M score**  **NT-proBNP (per 100 increment)** | 0.794  0.357 | 0.216  0.159 | -0.197  1.211 | 2.212 (1.326-3.692)  1.428 (1.046-1.950) | 0.002  0.025 | 2 | 41.349 | 28 | 45.829 |
| **IPSS-MC score (exact)‡** | 0.988 | 0.289 | 0.000 | 2.685 (1.524-4.731) | 0.0006 | 1 | 41.566 | 28 | 43.720 |

† Derived from IPSS-M / NT-proBNP multivariate model as log_2_[(TTP-related HR attributable to IPSS-M score)(TTP-related HR attributable to NT-proBNP)]=log_2_exp{0.508[IPSS-M score-(-0.197)]+0.294[(NT-proBNP/100)-1.211]}

‡ Derived from IPSS-M / NT-proBNP multivariate model as log_2_[(OS-related HR attributable to IPSS-M score)(OS-related HR attributable to NT-proBNP)]=log_2_exp{0.794[IPSS-M score-(-0.197)]+0.357[(NT-proBNP/100)-1.211]}

**Supplemental Figure 1.**KM curves confirmed the prognostic ability of the proposed GDF-15 cut-off (3727 ng/L) for both OS and TTP.

| 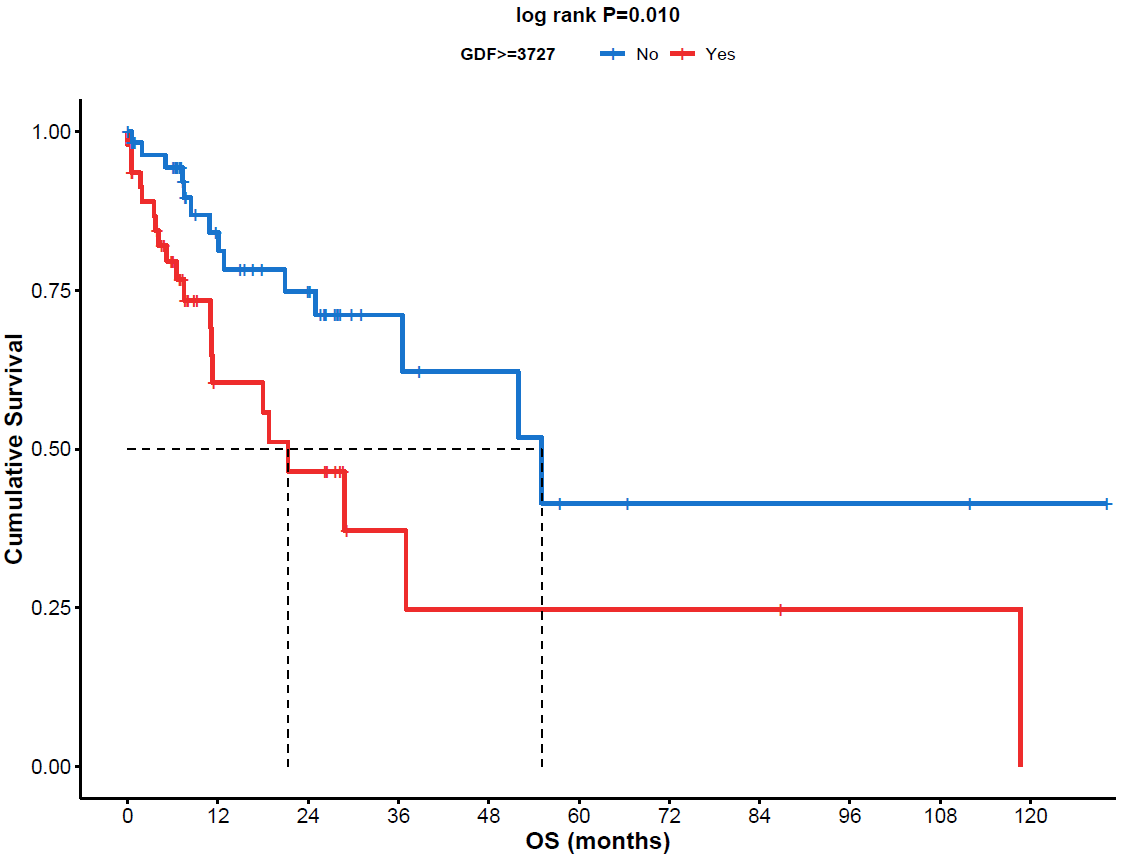 |
| --- |
| 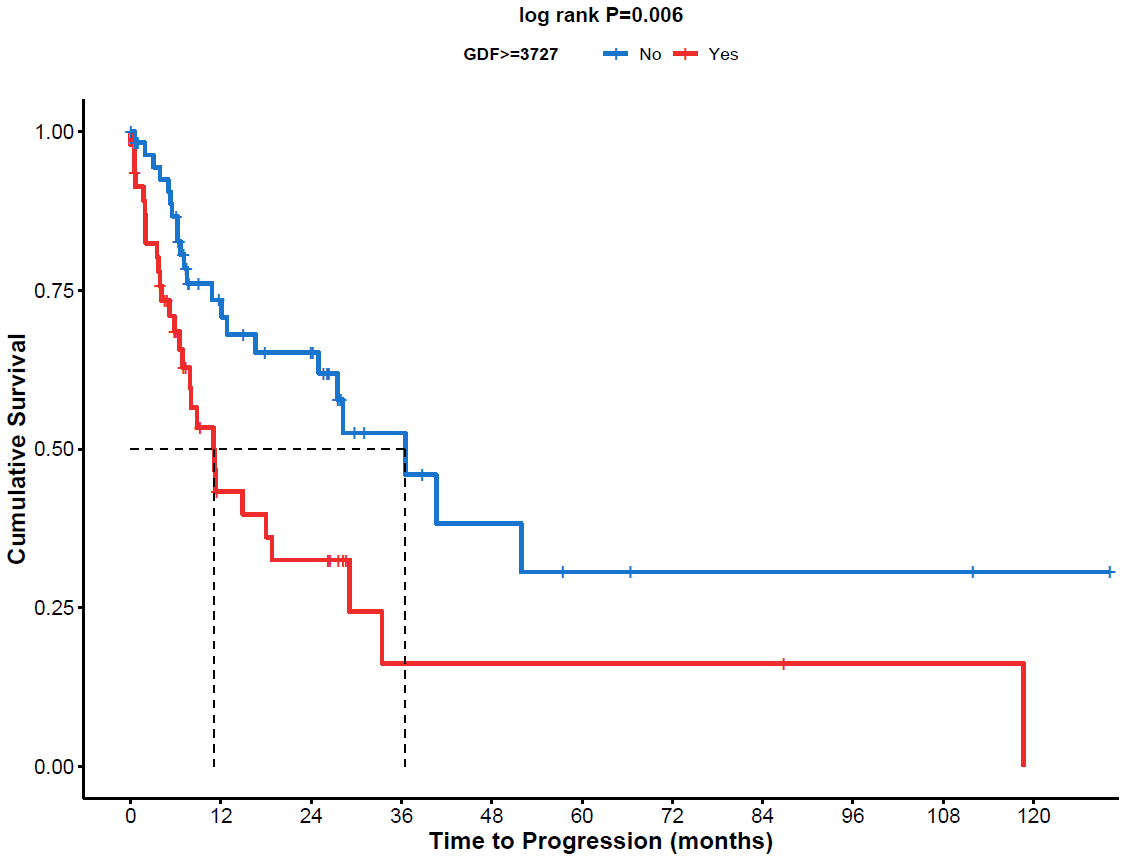 |

**Supplemental Figure 2.** ΚΜ curves based on the best NT-proBNP cut-off to serve as prognosticator (175 pg/L) for OS.


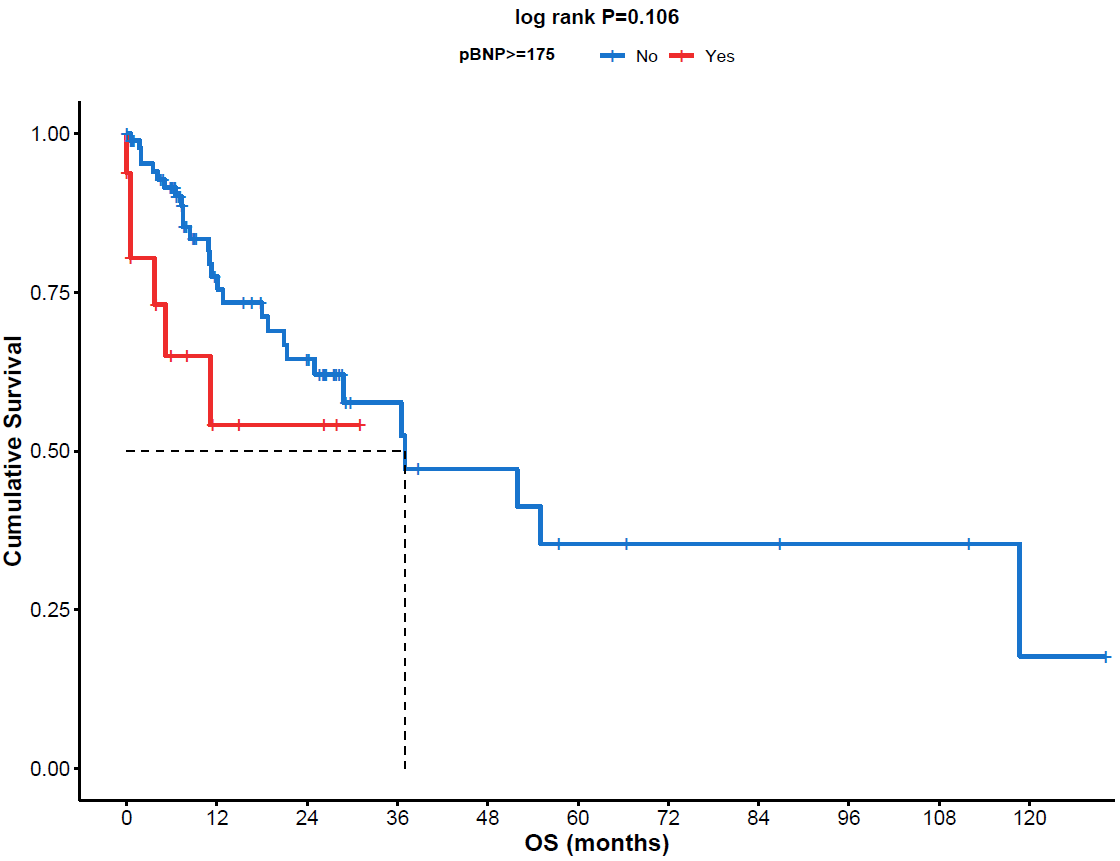


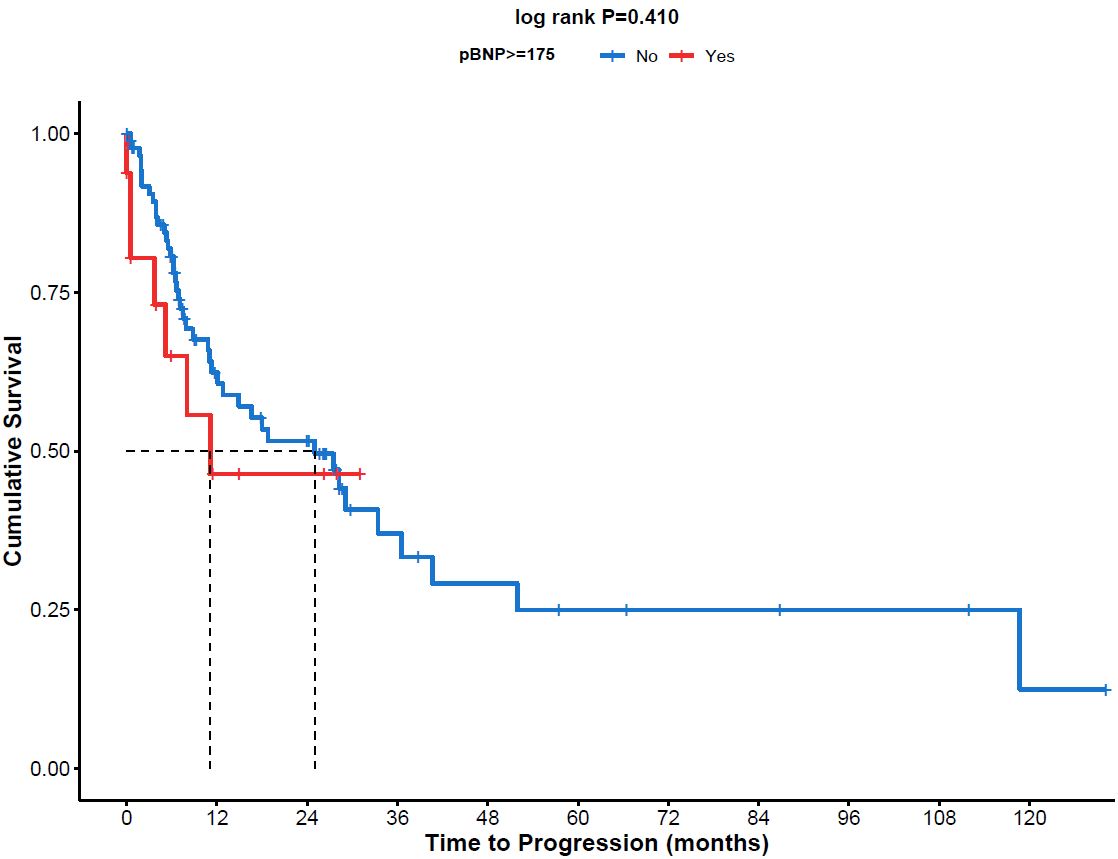


**References**

1. Elsa Bernard, Heinz Tuechler, Peter L. Greenberg, et al. Molecular International Prognostic Scoring System for Myelodysplastic Syndromes. *New England Journal of Medicine Evidence*. 2022;1(7).

2. Lamprianidou E, Kordella C, Kazachenka A, et al. Modulation of IL-6/STAT3 signaling axis in CD4+FOXP3- T cells represents a potential antitumor mechanism of azacitidine. *Blood Adv*. 2021;5(1):129-142.

3. Malcovati L, Crouch S, De Graaf A, et al. Mutation Profiles Identify Distinct Clusters of Lower Risk Myelodysplastic Syndromes with Unique Clinical and Biological Features and Clinical Endpoints. *Blood*. 2020;136(suppl 1):29.
